# The use of a geographic information system to identify a dairy goat farm as the most likely source of an urban Q-fever outbreak

**DOI:** 10.1186/1471-2334-10-69

**Published:** 2010-03-16

**Authors:** Barbara Schimmer, Ronald ter Schegget, Marjolijn Wegdam, Lothar Züchner, Arnout de Bruin, Peter M Schneeberger, Thijs Veenstra, Piet Vellema, Wim van der Hoek

**Affiliations:** 1 Centre for Infectious Disease Control, National Institute for Public Health and the Environment, A van Leeuwenhoeklaan 9, PO Box 1, 3720 BA Bilthoven, the Netherlands; 2 Municipal Health Service 'Brabant Zuidoost', Stadhuisplein 2, 5611 EM Eindhoven, the Netherlands; 3 Laboratory for Pathology and Medical Microbiology, De Run 6250, 5504 DL Veldhoven, the Netherlands; 4 Region East, Food and Consumer Product Safety Authority, De Stoven 22, Postbus 202, 7200 AE Zutphen, the Netherlands; 5 Department of Medical Microbiology and Infection Control, Jeroen Bosch Hospital, Tolbrugstraat 11, PO Box 90153, 5200 ME, 's Hertogenbosch, the Netherlands; 6 Department of Small Ruminant Health, Animal Health Service, Arnsbergstraat 7, PO Box 9, 7400 AA Deventer, the Netherlands

## Abstract

**Background:**

A Q-fever outbreak occurred in an urban area in the south of the Netherlands in May 2008. The distribution and timing of cases suggested a common source. We studied the spatial relationship between the residence locations of human cases and nearby small ruminant farms, of which one dairy goat farm had experienced abortions due to Q-fever since mid April 2008. A generic geographic information system (GIS) was used to develop a method for source detection in the still evolving major epidemic of Q-fever in the Netherlands.

**Methods:**

All notified Q-fever cases in the area were interviewed. Postal codes of cases and of small ruminant farms (size >40 animals) located within 5 kilometres of the cluster area were geo-referenced as point locations in a GIS-model. For each farm, attack rates and relative risks were calculated for 5 concentric zones adding 1 kilometre at a time, using the 5-10 kilometres zone as reference. These data were linked to the results of veterinary investigations.

**Results:**

Persons living within 2 kilometres of an affected dairy goat farm (>400 animals) had a much higher risk for Q-fever than those living more than 5 kilometres away (Relative risk 31.1 [95% CI 16.4-59.1]).

**Conclusions:**

The study supported the hypothesis that a single dairy goat farm was the source of the human outbreak. GIS-based attack rate analysis is a promising tool for source detection in outbreaks of human Q-fever.

## Background

Q-fever, a zoonosis caused by the bacterium *Coxiella burnetii*, is an emerging public health problem in the Netherlands. In May 2007, the first community Q-fever outbreak was documented around a single village in the province of Noord Brabant. A case control study carried out subsequently suggested a role of warm, dry weather conditions in addition to a residence location close to ruminant farms [1].

The unprecedented magnitude (>3000 notified human cases) and geographical spread within the province during the following years (2008-2009) suggested multiple sources of infection [2]. The affected area matches with the part of the Netherlands that has the highest concentration of dairy goat farms, some of which are very large (>5000 goats) [3]. From 2005 to 2008, clinical Q-fever represented by abortions or stillbirths in small ruminants, was diagnosed at 22 large (>50 goats) commercial dairy goat farms and 2 dairy sheep farms, mainly in the affected province [4]. Although large dairy goat farms were implicated, questions about exact transmission routes and risk factors remained unanswered.

In May 2008 the municipal health service (MHS) in the southeast region of the affected province was alerted by a cluster of human Q-fever cases in an urban area (88,000 inhabitants). Based on the distribution of cases in time and place, a common source was suspected. The MHS had been informed by the Animal Health Service that a dairy goat farm was diagnosed with Q-fever in their region. Some patients that were interviewed by the MHS indicated a pet farm in their neighbourhood as a potential source.

The objective of the study was to assess whether the cluster of human Q-fever cases in this urban area could be linked to the suspected source on the basis of the distribution of illness onset and residence location relative to small ruminant farms. Furthermore, the study explored the usefulness of a generic geographic information system (GIS) for source detection in the still evolving major epidemic of Q-fever in the Netherlands.

## Methods

### Human Q-fever cases

Q-fever is a notifiable disease in the Netherlands and the regional MHS was responsible for the outbreak investigation. A regional laboratory provided the majority of microbiological diagnostic services for the inhabitants of the MHS region. Submitted serum samples were screened with a *C. burnetii *complement fixation test (CFT, Siemens, the Netherlands). Notification criteria for acute Q-fever were a clinical presentation with at least fever, or pneumonia, or hepatitis and a fourfold rise in titre in the CFT or a single high titre or a high titre in two samples without a fourfold increase (CFT titre ≥1:4). All persons notified to the Dutch national surveillance system for infectious diseases (OSIRIS) who were residing or had visited the MHS region of Brabant Southeast and who had an illness onset from week 16 (14 April 2008) to week 32 (10 August 2008) were included.

A hypothesis generating questionnaire was used to get insight into possible sources of *C. burnetii *infection. Questions pertained to age, sex, day of illness onset, self-reported symptoms and complications, and a range of potential risk factors, such as travel history and occupational exposure. A recall period of four weeks before illness onset was used to investigate if cases had had relevant exposures immediately before the incubation period. Questionnaires to all notified cases were administered by trained public health employees from the MHS during house visits, or self-administered by mail.

Data on human cases was collected as part of the routine system of infectious disease surveillance and outbreak control. Because no additional information or laboratory materials were collected from patients, a medical ethical review was not needed.

### Meteorological data

We determined the predominant wind direction by day and calculated the number of days that the wind had blown in every wind direction during the estimated exposure period. These data were available for weather station Volkel (station 375 of the Royal Netherlands Meteorological Institute), located within 15 kilometres from the centre of the cluster area.

### Veterinary data

Veterinary data were obtained from the Animal Health Service. A mandatory veterinary notification system was only introduced on 12 June 2008 (week 24). If a veterinary source was suspected based on the exposure information from patients, the MHS notified the Food and Consumer Product Safety Authority.

### Data analysis

Residence locations of all Q-fever cases were plotted as point data on a digital map of the area. The Animal Health Service provided the locations and specifications of goat and sheep farms that were located within 5 kilometres of the average longitudinal and lateral coordinate of the cases, thus defining the centre of the cluster. The residential addresses of the cases was considered a proxy for level of exposure to small ruminant farms. We hypothesized that the attack rate would be higher for persons living close to the source and would decrease with increasing distance. Around each farm with more than 40 small ruminants we made concentric rings each expanding with a 1 kilometre radius (range 0-5000 meters) and a reference ring with a range from 5000 - 10,000 meters). Attack rates for residents living within these zones were calculated using a digital map of population distribution. Relative risks for 5 zones were estimated using the 5000-10,000 meters zone as reference. Mapping and spatial analyses were done with ArcGis 9 software (ESRI, Redlands, CA, USA).

## Results

Between week 16 and 33 (14 April-15 August 2008) the MHS Brabant Southeast notified 96 Q-fever cases. In 55 patients positive CFT titres were found in two sequential (≥14 days interval) samples (of which 42 cases had a fourfold increase in titre) and 41 patients had a single positive CFT titre. The median age of cases was 53 years (interquartile range 42-61) and the male to female ratio was 2.3:1. Day of onset of illness was known for 95 cases (median 4 June 2008). Nineteen individuals (20%) were hospitalised.

Eighty one (84%) of the 96 cases completed a questionnaire. Most commonly reported symptoms were fever >38°C (95%), fatigue (83%), headache (70%), night sweats (62%), dyspnoea (53%), malaise (52%), and myalgia (51%). The majority of cases (79%) presented clinically with a pneumonia. Five patients (6%) presented with hepatitis. Seventy eight (81%) of the 96 cases that were notified in the MHS region Brabant Southeast resided in one city with 87,752 inhabitants and a surface area of 54.56 km^2 ^(1608/km^2^). Eight cases were from adjacent municipalities and 10 from more distant municipalities and none of these reported to have a work address within the city.

**Figure 1:**
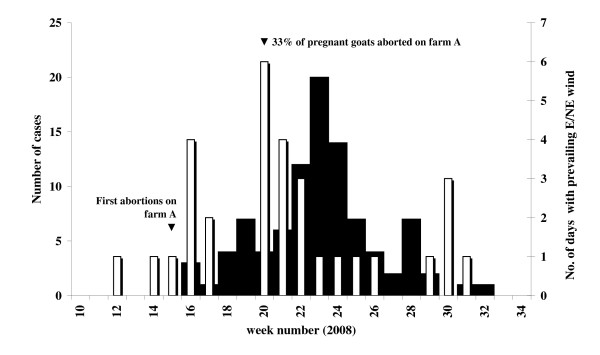
**Epidemic curve of Q-fever and wind direction**. Number of Q-fever cases in the Municipal Health Service region Brabant Southeast by week of illness onset (n = 95, black bars); and number of days in the week with prevailing eastern or north-eastern wind (white bars). Information on date of illness onset is missing for one case.

We estimated from the epidemic curve (Figure 1) that the likely period of infection was from week 15 (mid-April) to week 24 (mid-June). This encompassed the known two to four week incubation period for *C. burnetii *and was based on date of symptom onset for 95 of the 96 cases.

### Veterinary investigations

**Figure 2:**
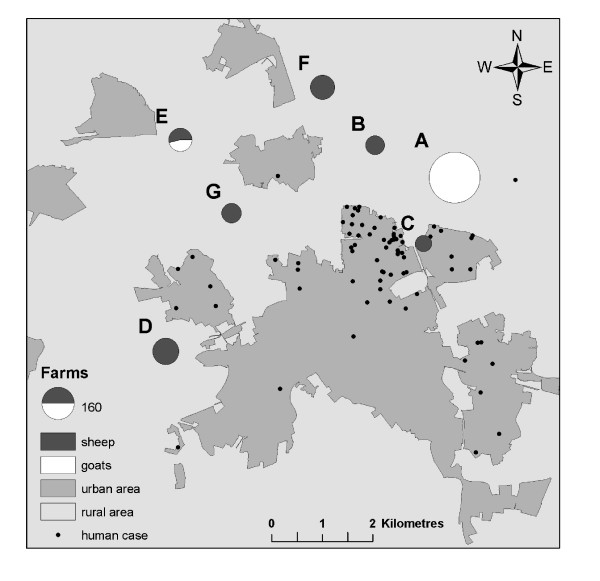
**Map of the study area**. Locations of goat and sheep farms with >40 animals (farm A-G) and residential addresses of Q-fever cases in urban and rural areas, 14 April to 10 August 2008.

There were 60 locations with small ruminants within the 5 kilometre zone around the centre of the human Q-fever cluster. Seven of these locations were large farms (A-G) with more than 40 small ruminants, including one large dairy goat farm (A, >400 goats), one mixed sheep/goat farm (E, 126 goats and 90 sheep) and 5 sheep farms (B, C, D, F, and G with between 57 and 118 sheep). These farms are mapped in figure 2. Farm A reported an abortion wave on 21 April 2008 (week 17) to the Animal Health Service and submitted two aborted lambs for post mortem examination. *C. burnetii *infection was confirmed in placental tissue from aborted goats by immunohistochemistry. The first abortions were noticed in week 15 (mid April) 2008. No other farms in the area reported abortions in the weeks prior to the outbreak. At the time of the visit by the Animal Health Service on 15 May (week 20), 40 out of 120 pregnant goats had aborted (33%), 20 (17%) had an uncomplicated pregnancy and got healthy offspring, while another 60 animals still had to deliver. No veterinary problems were reported by the other farms. A pet farm in the part of the city where most of the cases resided was visited by the Food and Consumer Product Safety Authority. Three tested goats and one sheep had a weak positive PCR (only one of three *Coxiella *specific genomic targets positive). However these animals had not been pregnant recently. Hygiene standards at the pet farm were good and none of the employees of the pet farm developed symptoms or had been diagnosed with acute Q-fever. It was concluded that these animals were not the main source of the large cluster of human cases.

### Meteorological data

With an east to north-eastern wind most of the city lies downwind of the dairy goat farm and could potentially have been exposed to contaminated dust particles. During the study period these wind conditions were common in week 16 (14-20 April) with 4 out of 7 days and in week 20 (12-18 May) with 6 out of 7 days (Figure 1).

### GIS analysis

Distance-related attack rates and relative risks for increasingly larger sequential ring buffers from each of the 7 large farms (A-G) are shown in Table 1. A gradual diminishing relative risk with increasing distance was observed for farm A, the large dairy goat farm with the reported abortion wave, and two small sheep farms B and C.

**Table 1:** Attack rates of acute Q-fever among residents within circular distance rings around each potential source (A-G) expressed per 100,000 persons over week 16-32 (14 April-10 August 2008), with relative risks (RR) and 95% confidence intervals (95% CI)

Distance from source	A	B	C	D	E	F	G
***0-1 kilometre***							
Attack rate^1^	0	0	413	178	0	0	0
RR^2 ^(95% CI)	0	0	53 (24-114)	8 (1-61)	0	0	0
Q-fever cases	0	0	35	1	0	0	0
Population	92	136	8482	562	119	281	1444

***0-2 kilometre***							
Attack rate	376	352	203	46	0	13	76
RR (95% CI)	31 (16-59)	25 (13-49)	26 (12-54)	2 (1-5)	0	1 (0-5)	5 (2-13)
Q-fever cases	33	27	59	6	0	1	8
Population	8788	7671	29,098	13,022	9852	7632	10,582

***0-3 kilometre***							
Attack rate	241	228	137	28	19	69	102
RR (95% CI)	20 (11-36)	16 (9-30)	18 (8-36)	1 (1-3)	1 (0-2)	4 (2-8)	7 (4-13)
Q-fever cases	59	58	69	9	4	12	28
Population	24,461	25,487	50,349	32,709	20,521	17,506	27,329

***0-4 kilometre***							
Attack rate	124	133	101	26	60	147	98
RR (95% CI)	10 (6-19)	10 (5-17)	13 (6-27)	1 (1-2)	2 (1-4)	8 (5-13)	7 (4-12)
Q-fever cases	70	66	71	15	19	49	62
Population	56,585	49,570	70,151	58,002	31,425	33,270	63,547

***0-5 kilometre***							
Attack rate	92	88	86	53	94	100	79
RR (95% CI)	8 (4-14)	6 (3-11)	11 (5-23)	3 (2-4)	4 (3-6)	5 (3-9)	6 (3-10)
Q-fever cases	71	74	78	51	54	71	72
Population	77,558	84,374	90,779	96,402	57,329	70,680	91,123

***>5-10 kilometre***							
Attack rate	12	14	8	21	25	18	14
RR	1	1	1	1	1	1	1
Q-fever cases	13	13	8	39	40	21	17
Population	107,680	93,469	102,192	185,269	162,141	113,966	121,978

^1 ^Attack rate: number of Q-fever cases per 100,000 population during the outbreak

^2 ^RR = relative risk, attack rate divided by the attack rate of the reference category 5-10 km, with 95% confidence interval (CI)

Farm A and B both showed a gradual diminishing relative risk with increasing radius, with the exception of the first 1-kilometer zone, possibly due to the small denominator. There were no significant differences in attack rates between farm A and B. The attack rates within 2 and within 3 kilometres were significantly higher for farm A than for farm C (p = 0.004 and p = 0.001 respectively). The highest attack rate for farm A was for inhabitants residing in the south to south-western direction within 2 kilometres of the farm. Persons living in this zone had a much higher risk for Q-fever than those living more than 5 kilometres away (Relative risk 31.1 [95% CI 16.4-59.1]). In the 4-5 kilometre zone from farm A, only 1 case was added and the risk of Q-fever for people living in this zone was very low. The other farms D, E, F, G had low attack rates close to the farms compared to farms A-C and they did not show a gradual diminishing relative risk with increasing distance and were considered unlikely infection sources.

## Discussion

Within an on-going large epidemic of Q-fever in a province in the south of the Netherlands we identified a distinct urban cluster of notified Q-fever cases. We combined epidemiological data on notified cases, veterinary and meteorological data in a generic geographic information system to analyse this cluster. The study showed that living within 2 kilometres of a dairy goat farm with abortion problems posed a high risk for Q-fever. Furthermore, the time period between the duration of the abortion wave on this dairy goat farm and illness onset in the human cases suggests that airborne transmission of *C. burnetii *from the dairy goat farm could have been the cause of this outbreak. This was further supported by predominant easterly winds on a number of days that could have taken contaminated dust particles to the people living southwest from the farm.

Findings from this urban outbreak are consistent with those from the first community Q-fever outbreak in the Netherlands that occurred in a rural area in 2007. In the 2007 outbreak, airborne transmission from nearby small ruminant farms located to the northeast of the village was suspected as the main route, facilitated by a predominant wind direction from the east during a period of extreme dry weather [1].

There are reports from the international literature that *C. burnetii *particles can spread from farmland over long distances, often facilitated by strong winds towards urban areas [5]. In a community outbreak in the United Kingdom cases were identified up to 18 kilometres from the source [6]. Our results suggest a low risk beyond 5 kilometres from the infection source. The role of wind speed and direction is difficult to analyse when a farm is infectious for a longer period of time.

### Source detection

In the high incidence area in the south of the Netherlands there are thousands of locations with goats and sheep in addition to the many cattle, pig and poultry farms. In this environment the investigation and sampling of all potential animal infection sources to explain human clusters is impossible. Goats, sheep and cattle on many farms throughout the country show serological evidence of previous infection with *C. burnetii*. However, based on the experience since 2007, the prevailing opinion among the human and veterinary public health community is that abortion waves at large dairy goat farms play the predominant role in transmission [4]. Abortion rates of more than 5% on large dairy goat and sheep farms have to be notified since June 2008. However, abortions in deep litter houses with many hundreds of animals might easily go unnoticed. Attack rate analysis as proposed in the present paper could be an additional tool for source detection.

In order to apply this method real-time, detailed geographic information with residence locations of cases and farm locations, and early notification of human clusters by health professionals are required. The complement fixation test is known to be specific but definitive diagnosis may be delayed because often paired samples are required [7]. Increased awareness among patients and physicians, as well as introduction of rapid laboratory assays, such as PCR, may further reduce the delay between onset of illness and diagnosis and notification [8]. Moreover, in June 2008 Q-fever became a veterinary notifiable disease. This legal framework has facilitated communication between the human and veterinary public health sectors. Clearly, it is essential that the MHS can alert general practitioners in a region where there is a small ruminant farm with clinical Q-fever. Veterinary information on Q-fever status of farms in the near proximity, for example clinical Q-fever or positive bulk milk status, could facilitate to pinpoint the most likely sources in an exposed area.

The analysis of the 2008 urban cluster was facilitated by the relatively small number of farms in the area. Even so, the attack rate analysis showed similar results for three farms (A-C) that were located very close to each other. The tool can provide only a rough indication but could facilitate efficient source detection by placing animal locations in classes of 'possible' and 'unlikely'. This would have to be followed by a veterinary risk assessment and possible sampling at suspected farms. Generating the attack rates requires experience in the use of GIS software. When adequate tools for GIS analyses are available, the workload is limited and the procedure can be automated.

### Limitations of the study

This retrospective cohort study was performed in a new endemic area for Q-fever in the Netherlands where dairy goat farming started recently. Q-fever was laboratory confirmed on the newly established dairy goat farm but no systematic environmental sampling took place in the cluster area. Therefore we cannot exclude that other sources might have been present in the same time period in the region.

Most Q-fever infections remain asymptomatic or give aspecific signs and symptoms. Differences in diagnostic testing and alertness of general practitioners might have led to ascertainment bias. To provide a reliable estimate of the attack rate all patients with symptoms compatible with Q-fever would have to be tested or population surveys would have to be done to include asymptomatic cases.

### Research priorities

Conclusive evidence of a link between certain animals and human Q-fever cases can only be provided by genetic analysis of human, animal and environmental samples. Current typing techniques provide insufficient contrast and methods for culturing and sequencing carry bio safety concerns and are still under development. The presented attack rate analysis assumes homogeneity in the distribution of *Coxiella *in each concentric ring. In future analyses landscape features should be taken into account to make a more informative analysis possible. This should be combined with information on movement patterns of human cases, recreational activities and other behavioural factors that might change the risk of exposure.

## Conclusions

We found a clear epidemiological link between a cluster of human Q-fever cases and a Q-fever positive dairy goat farm. The present study suggests an effective range of airborne *C. burnetii *spread of <5 kilometres. GIS-based automated attack rate analysis is a promising tool that could classify animal locations as possible or unlikely infection sources. This methodology should be transformed from a retrospective analysis to a real-time pinpointing tool to guide environmental sampling of potential sources. This requires close collaboration between the human and veterinary public health sectors to ensure timely detection of cases, identification of plausible sources and standardised environmental sampling, and application of public health and veterinary preventive measures.

## Competing interests

The authors declare that they have no competing interests.

## Authors' contributions

BS, PV and WvdH were involved in designing the study and the protocol for data collection. RtS, MW, LZ, AdB and PV provided the human, veterinary and environmental data. BS and TV conducted the data analysis. TV produced the map. BS produced the first draft of the paper, which was revised by WvdH and PS following contributions from RtS, MW, LZ, AdB, TV and PV. All authors read and approved the final manuscript.

## Pre-publication history

The pre-publication history for this paper can be accessed here:

http://www.biomedcentral.com/1471-2334/10/69/prepub

## References

[B1] Karagiannis I Schimmer B van Lier A Timen A Schneeberger P van Rotterdam B de Bruin A Wijkmans C Rietveld A van Duynhoven Y Investigation of a Q fever outbreak in a rural area of The Netherlands Epidemiol Infect 2009 137 1283 1294 10.1017/S0950268808001908 19161644

[B2] Schimmer B Morroy G Dijkstra F Schneeberger PM Weers-Pothoff G Timen A Wijkmans C Hoek W van der Large ongoing Q fever outbreak in the south of The Netherlands, 2008 Euro Surveill 2008 13 pii: 18939 18761906

[B3] Schimmer B Dijkstra F Vellema P Schneeberger PM Hackert V ter Schegget R Wijkmans C van Duynhoven Y Hoek W van der Sustained intensive transmission of Q fever in the south of the Netherlands, 2009 Euro Surveill 2009 14 pii: 19210 10.2807/ese.14.19.19210-en 19442401

[B4] Brom R Van den Vellema P Q fever outbreaks in small ruminants and people in the Netherlands Small Ruminant Res 2009 86 74 79 10.1016/j.smallrumres.2009.09.022

[B5] Tissot-Dupont H Amadei MA Nezri M Raoult D Wind in November, Q fever in December Emerg Infect Dis 2004 10 1264 1269 15324547 10.3201/eid1007.030724 PMC3323349

[B6] Hawker JI Ayres JG Blair I Evans MR Smith DL Smith EG Burge PS Carpenter MJ Caul EO Coupland B Desselberger U Farrell ID Saunders PJ Wood MJ A large outbreak of Q fever in the West Midlands: windborne spread into a metropolitan area? Commun Dis Public Health 1998 1 180 187 9782633

[B7] Péter O Dupuis G Peacock MG Burgdorfer W Comparison of enzyme-linked immunosorbent assay and complement fixation and indirect fluorescent-antibody tests for detection of Coxiella burnetii antibody J Clin Microbiol 1987 25 1063 1067 3298311 10.1128/jcm.25.6.1063-1067.1987 PMC269137

[B8] Schneeberger PM Hermans MHA van Hannen EJ Schellekens JJA Leenders ACAP Wever PC Real-time PCR on serum samples is indispensable for early diagnosis of acute Q-fever Clin Vaccine Immunol 2010 17 286 290 10.1128/CVI.00454-09 20032219 PMC2815520

